# Gene Mutations Associated with Temporomandibular Joint Disorders: A Systematic Review

**DOI:** 10.4236/oalib.1101583

**Published:** 2015-06-03

**Authors:** Dhruvee Sangani, Akiko Suzuki, Helena VonVille, James E. Hixson, Junichi Iwata

**Affiliations:** 1 Department of Epidemiology, Human Genetics & Environmental Sciences, The University of Texas School of Public Health, Houston, TX, USA; 2 Department of Diagnostic & Biomedical Sciences, The University of Texas Health Science Center at Houston School of Dentistry, Houston, TX, USA; 3 Center for Craniofacial Research, The University of Texas Health Science Center at Houston School of Dentistry, Houston, TX, USA; 4 The University of Texas School of Public Health Library, Houston, TX, USA; 5 The University of Texas Graduate School of Biomedical Sciences at Houston, Houston, TX, USA

**Keywords:** Temporomandibular Joint, Temporomandibular Joint Disorders, Systematic Review

## Abstract

**Background:**

The temporomandibular joint (TMJ) is a bilateral synovial joint between the mandible and the temporal bone of the skull. TMJ disorders (TMDs) are a set of complicated and poorly understood clinical conditions, in which TMDs are associated with a number of symptoms including pain and limited jaw movement. The increasing scientific evidence suggests that genetic factors play a significant role in the pathology of TMDs. However, the underlying mechanism of TMDs remains largely unknown.

**Objective:**

The study aimed to determine the associated genes to TMDs in humans and animals.

**Methods:**

The literature search was conducted through databases including Medline (Ovid), EMBASE (Ovid), and PubMed (NLM) by using scientific terms for TMDs and genetics in March 2015. Additional studies were identified by searching bibliographies of highly relevant articles and Scopus (Elsevier).

**Results:**

Our systematic analyses identified 31 articles through literature searches. A total of 112 genes were identified to be significantly and specifically associated with TMDs.

**Conclusion:**

Our systematic review provides a list of accurate genes associated with TMDs and suggests a genetic contribution to the pathology of TMDs.

## 1. Introduction

The temporomandibular joint (TMJ; *aka* the jaw joint) is a bilateral synovial joint between the mandible and the temporal bone. The TMJ consists of an articular disc located in the articular cavity, the glenoid fossa of the temporal bone and the mandibular condyle, and is filled with synovial fluid. The TMJ is unique because it consists of two joints in one bone, which is required to work simultaneously, and it acquires two major movements of the mandibular condyles: hinge and sliding, which is important for smoothly opening/closing mouth and chewing. TMJ disorders (TMDs) are a sub-classification of musculoskeletal disorders that result from stomatognathic system defects, and affect the function of masticatory muscles, the TMJ, and its associated structures [1].

TMDs are a set of complicated and poorly understood clinical conditions, which are associated with a number of symptoms including pain and limited jaw movement. The estimated prevalence of TMDs ranges from 5% to 60% worldwide [2]. TMDs occur as an individual disorder or associated with other syndromes, and TMJ pain is the second most common musculoskeletal pain after chronic low back pain [3] [4]. Since the symptoms of TMDs include pain, inflammation, and limited jaw movements, the etiology of TMDs may be complex. In addition, the susceptibility to TMDs may not be equal; for example, it is known that women are more susceptible than men. Severe and/or chronic cases of TMDs often occur with pain, but in mild cases, pain may be resolved over time. The possible influence factors of TMDs are mechanical and/or psychic stresses, hormones, genes, ethnicity, social status, and gender [5]. It has been postulated that women and adolescents have a higher risk for TMDs, compared to men [6] [7]. TMJ osteoarthritis (OA) is also diagnosed at the later stages of severe cases of TMDs [8]. The extent of causative genes of TMDs has been of prime interest [9]. The increasing scientific evidence suggests that genetic factors may play a significant role in the pathology of TMDs [10] [11]. However, the underlying mechanism of TMDs remains largely unknown. In this study, we performed a systematic review to identify genes associated with TMDs.

Currently, there are a few dental experts trained to precisely diagnose and treat this complex disorder. The treatment of TMDs has been very broad and general, including medications such as non-steroidal anti-inflammatory drugs (NSAIDs), muscle relaxants, physiotherapy, splints, biofeedback, and cognitive behavioral therapy. This study aims to provide precise information and highlight scientific literatures identifying genes associated with specific TMD conditions. Since not much research has been done on humans, animal studies have also been included in this systematic review. With knowledge of genetic studies, physicians and dentists can make diagnosis, tailor treatments, and predict susceptibility among people.

## 2. Methods and Materials

### 2.1. Eligibility Criteria for Systemic Review

The PRISMA (Preferred Reporting Items for Systematic reviews and Meta-Analyses) guideline and checklist was followed for this systematic review. Articles included in our systematic review met the following eligibility criteria: 1) described genes that are identified in TMDs; 2) were published as original articles (not as review articles, editorials, or comments); 3) were published in the English language; 4) were published between the years 2000 and 2015; and 5) specified TMD caused by gene mutations. Some articles were excluded from our systematic review because of one or more of the following reasons: a) gene mutations were not described in original articles; b) the type of TMDs (signs, symptoms, etc.) was not described or not diagnosed; c) the symptom was only pain, or the other symptoms and/or evaluations were not described; d) TMDs resulted from environmental factors; e) the studies had been done in other joints of the body; and f) others that include articles that failed to fit in any of the above criteria.

### 2.2. Information Sources and Search

The online databases that were searched included: Medline (Ovid), PubMed (National Library of Medicine), and EMBASE (Ovid). In addition, highly relevant citations were searched in Scopus (Elsevier) to determine if any unique studies were missed by the database searches. Bibliographies of highly relevant articles were also examined. The Primary Excel Workbook for Systematic Reviews was used to track all search strategies and results, as described previously [12]. All search strategies are listed in full in Table S1 and Table S2. The search was limited to studies from 2000 through 2015 as genetic studies have become more technologically advanced in recent years, and very few genetic studies on TMDs were conducted before the year 2000.

### 2.3. Study Selection and Data Collection

RefWorks (Proquest) was used to store all citations found during the entire search process. The Primary Excel Workbook for Systematic Reviews (VonVille, Helena M. Primary Excel Workbook for Systematic Reviews, http://libguides.sph.uth.tmc.edu/excel_SR_workbook) was used to screen titles and abstracts of items found through database search. Titles and abstracts were screened by the first author (DS). The full text of articles not excluded was retrieved and reviewed by the first and second authors (DS, AS) with all data related to both screening and reviewing recorded in the Primary Excel Workbook. The data collected were displayed as a descriptive narrative. A codebook for data extraction from eligible articles was developed. The data elements that were extracted for the codebook included citation information, study level information (characteristics and results), and quality level information.

## 3. Results and Discussions

**Figure 1:**
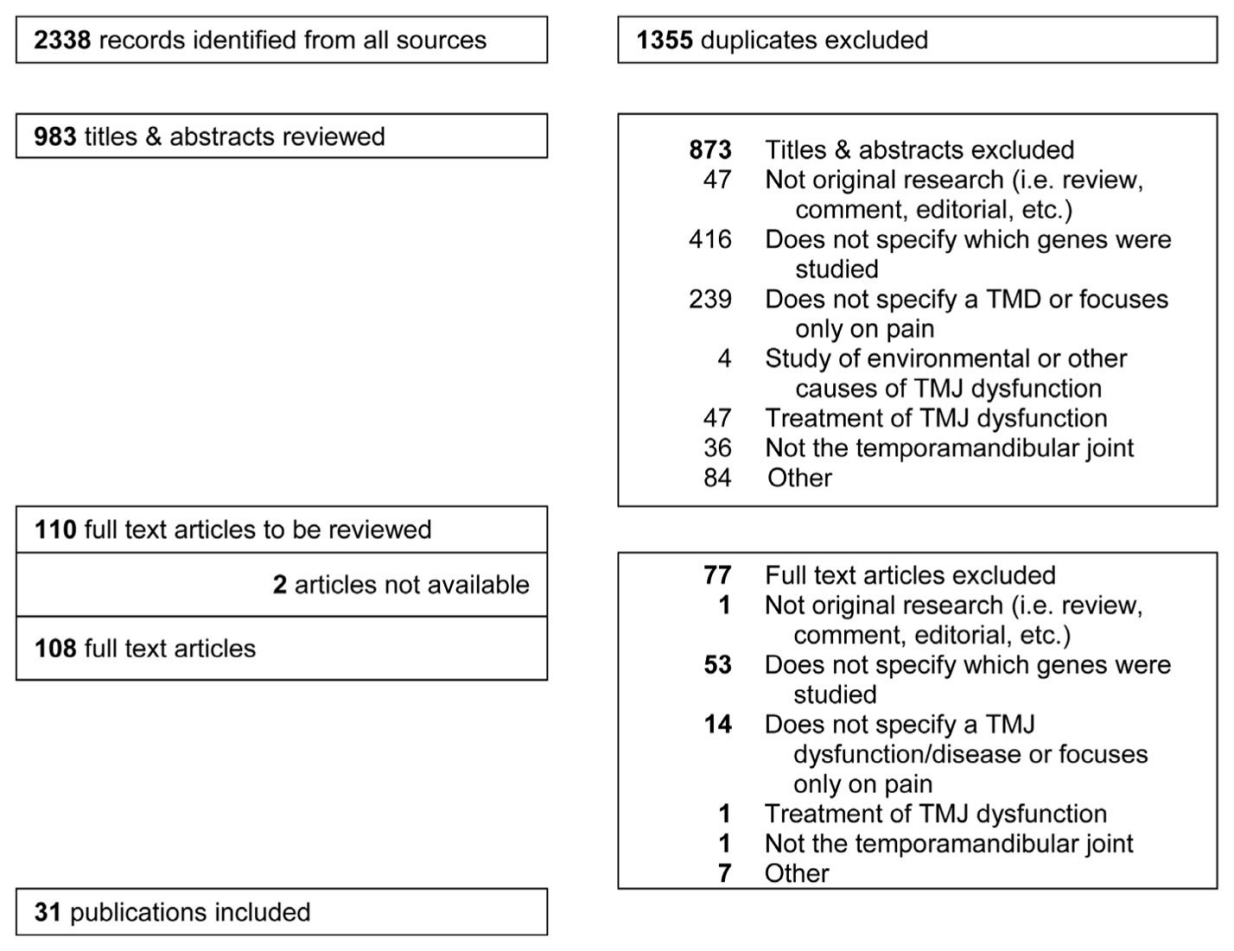
Prisma flowchart for selection of articles.

Nearly 900 unique articles were identified using the search strategies described above (see Figure 1 and Supplemental Information, Tables S1 and S2 for the database search strategies). Approximately 61% of the articles included were published after 2010. Interestingly, most of the articles included were published in 2014. Among the articles included for review (n = 31), 10 studies were done on humans while 21 studies were conducted on animal models (Table 1 and Table 2). One study conducted research on both mouse and human subjects [13]. 18 out of 31 articles conducted research on mice while 1 article studied in porcine, 2 articles in rats, and 1 article in rabbits. The articles from 2000–2015 covered most of the research related to genes in TMDs. Eight studies were conducted in 2014 and 2 studies were conducted in 2015. The demographic characteristics among the human studies were very different. Among the human studies, different ethnic groups such as Koreans, Japanese, Brazilian, Finnish, and Turkish populations were studied (Table 1). Three articles did not mention the ethnicity of the population studied [14] [15] [16]. One study indicated that in the miscegenetic Brazilian population, the age was significantly associated with TMDs [17]. Two studies were carried out only in women to determine the association of the estrogen receptor alpha (*ESR*1) gene with TMDs. One study [18] indicated the contribution of *ESR*1 on TMDs whereas another study [19] suggested no association of *ESR*1 with TMD symptoms such as clicks, crepitus, and bone erosion. Females are more affected by TMJ pain and degeneration due to the effects of *β*-estradiol on the TMJ [18]. Three studies show no statistically significant changes in candidate TMD causative genes: *MAOA* (monoamine oxidase-A), *ESR*1 (estrogen receptor alpha), and *TNF* (tumor necrosis factor alpha) [16] [19] [20]. Several polymorphisms have been described in the promoter region of MMP genes, and these polymorphisms modify gene expression and function of the MMPs. *MMP*1 (matrix metalloproteinase 1) polymorphism, but not *MMP*3 and *MMP*9 polymorphisms, is associated with osteophyte and erosion of the mandibular condyle. In the Brazilian *MMP*1 2G/2G homozygous subjects, the probability of developing degeneration of the mandibular condyle is 2.47 times higher than that in the *MMP*1 1G/2G and 1G/1G individuals [17]. *ADAMTS*5 (a disintegrin and metalloproteinase with thrombospondin motif 5) is involved in deformation of the articular disc associated with internal derangement and osteoarthritis in the TMJ [14]. Thus, proteinases contribute to degrade the articular disc and the surface of the mandibular condyle during the progression of TMDs. Homozygous polymorphism in *ANKH* (a human homolog of the murine progressive ankylosis gene, *ank*) more likely develops TMJ closed lock compared to controls [13]. The human leukocyte antigen (HLA) complex is a major histocompatibility complex (MHC) which is present on the surface of immune cells. Polymorphism in *HLA-DRB*1 (MHC class II, DR beta 1) allele is associated with erosion of the mandibular condyle [21]. These findings suggest that inflammation and autoimmune diseases may affect the progression of TMDs. Polymorphism in the fibroblast growth factor 2 (*FGF*2) gene is involved in the pathogenesis of TMJ synovial chondromatosis that is a rare proliferative disorder characterized by the formation of cartilaginous or osteocartilaginous nodules in synovium and articular cavities [15]. A recent genome-wide association study (GWAS) identified 22 independent loci showing association with degeneration of the mandibular condyle in East Asian populations. Among them, *TSPAN*9 (tetraspanin 9) polymorphism showed strongest association with TMD [22]. TSPAN9 mediates a signal transduction that plays a role in the regulation of cell development, activation, growth, and motility. Thus, some cell signaling pathways may be involved in the pathogenesis of TMDs. Although most of the studies have been done in small sample sizes, 28 out of 31 studies identified genes to be causal or associated with TMDs. This finding can lead us to believe that gene mutations are a causative factor for the incidence of TMDs.

In spite of extensive attempts, the molecular mechanism of TMDs is largely unknown because of the complex interaction of genes with other confounding factors like hormones, age, ethnicity, and environment. The presence of more than one symptom may be interdependent and act as a confounding factor [19]. Due to heterogeneity of symptoms, there are a lot of variations in diagnosis of TMDs worldwide. The appropriate treatments for TMD can only be initiated with a precise diagnosis. A number of imaging techniques including ultrasonography, computed tomography (CT), arthrography, and magnetic resonance imaging (MRI) have been adopted to confirm the diagnosis made by history taking and clinical examination. In 1992, The Research Diagnostic Criteria for Temporomandibular Disorders (RDC/TMD) was proposed as the basis for diagnosis of these complicated disorders [23]. In 2004, RDS/TMD has been replaced with Diagnostic Criteria for TMD (DC/TMD) because the validity of RDC/TMD was not sufficient enough in clinic [24]. Only 2 studies used the RDC/TMD diagnostic criteria, and none of the clinical studies have used the latest DC/TMD criteria yet.

This systematic review has much strength in identifying causative genes and their genetic interactions; however, it has a few limitations such as the year range of 2000–2015 and the English language. All the human studies had more number of females participating in the study. The participation of unequal numbers of gender might affect the validity of the results that women are more susceptible to TMDs. In addition, due to a broad classification and diagnostic criteria of TMDs, a potent detection bias cannot be ignored. Publication bias also can be the reason for limited literature being published. Future research with larger sample sizes will enable us to better understand genetic association with TMDs. Recent advances have introduced new techniques like GWAS that can help us discover genes associated with TMDs.

## 4. Conclusion

From a public health perspective, there is an urgent need to address TMDs and determine their causative factors. Despite different diagnostic criteria used, most of the included studies find associations between genes and TMDs. Future studies should incorporate objective diagnostic methods such as DC/TMD for TMD diagnosis to yield consistent results. Understanding the molecular mechanism is important to individualize treatment for TMDs in order to alleviate the overall burden of TMDs. Further studies to identify genes associated with TMDs will enable us to specifically diagnose TMDs and improve the quality of treatment. These findings will also help us understand why there is a gender disparity in TMDs and who may be more likely to suffer from TMDs.

## Supplementary Material



## Figures and Tables

**Table 1:** Human studies.

Author publication year country of study	Study population characteristics	Diagnosis diagnostic criteria	Gene(s) studied	Results	Study limitations
Planello [17] 2011 Brazil	Mean Age of Cases 42.82 ± 14.96 N = 115: F = NR, M = NR Mean Age of Controls 38.04 ± 14.17 N = 117: F = NR, M = NR Ethnic status: Brazilian	Osteophytes, erosion, avascular necrosis, subchondral cyst, loose bodies, OA CT, Interview, MRI	MMP1 MMP3 MMP9	Only MMP1 2G/2G genotype was found to be significant (P < 0.003). Effect of age on TMJ degeneration was significant (P < 0.002)	Small sample size to detect association of MMP3 gene
Huang [13] 2011 Japan	Mean Age of Cases 15.5 to 69.7 N = 55: F = 44, M = 11 Mean Age of Controls NR N = NR: F = NR, M = NR Ethnic status: Japanese	TMJ clicking (disc displacement with reduction) or closed lock (disc displacement without reduction) and not having previous history of trauma, neoplasm and/or surgery in the TMJ; TMJ clicking used as controls Clinical evaluation, MRI	ANKH	Two alleles of ANKH-OR polymorphism were identified. ANKH-OR homozygotes were more likely to develop closed lock compared to controls (P = 0.011)	Calculation of minimal sample size was unattainable, Low response rate could have resulted in sampling bias, application of cohort study would have explored the influence of age on TMD, high percentage of patients were unwilling to take a blood test for assessment, study failed to indicate equivalence between fibrous ankylosis in mice and closed lock in humans
Kim [19] 2010 South Korea	Mean Age of Cases 31.45 ± 15.25 years N = 74: F = 50, M = 24 Mean Age of Controls 35.58 ± 12.47 N = 64: F = 41, M = 23 Ethnic Status: Korean	MPDS, IDD with reduction, IDD without reduction, OA, Osteoarthrosis RDC/TMD	ESR1	No statistical difference between study group and control group (P > 0.5)	Small sample size
Ribeiro-Dasilva [18] 2009 Brazil	Age for Cases 18 – 44 N = 300: F = 300, M = 0 Age for Controls 31.1 ± 8.36 N = 100: F = 100, M = 0 Ethnic Status: White, Afro-American, Asiatic	Disc displacement with and without reduction, with limited mouth opening, without reduction and without limited mouth opening RDC/TMD	ESR1	Presence of [GC] haplotype in ER*α* gene was higher in TMD with pain compared to control group (P = 0.0028)	Small sample size, admixed Brazilian population limits generalizability of data, only data from articular pain included and not muscular pain
Matsumoto [14] 2008 Japan	Mean Age of Cases 50.11 N = 9: F = 8, M = 1 Mean Age of Controls 65.5 N = 2: F = 2, M = 0 Ethnic Status: NR	ID, OA NR	ADAMTS-5	ADAMTS-5 is involved in deformation of the TMJ discs with ID and OA (P < 0.01)	Small sample size, limited use of TMJ disc cells
Helenius [21] 2004 Finland	Mean Age of Cases 43.57 N = 84: F = 56, M = 28 Mean Age of Controls: NR N = 100: F = NR, M = NR Ethnic status: Finnish	RA, MCTD, AS, SPA ARA 1987 for RA, Alarcon-Segovia for MCTD, MNYC for AS, ESSG for SPA	HLA-DRB1	In the whole patient population, HLA-DRB1 allele was significantly associated with TMJ erosions (P = 0.0014)	Controls were not clinically or radiologically examined
Li [15] 2014 China	Mean Age of Cases NR N = 3: F = NR, M = NR Mean Age of Controls NR N = 3: F = NR, M = NR Ethnic status: NR	Synovial chondromatosis for cases; Open Reduction for controls NR	FGF2	FGF2 was involved in the pathogenesis of synovial chondromatosis (P < 0.01)	NR
Yamaguchi [22] 2015 Japan	Mean Age of Cases Japanese 25.6 Koreans 28.5 N of Japanese = 105: F = 93, M = 12 N of Koreans = 41: F = 23, M = 18 Mean Age of Controls Japanese 25.6 Koreans 28.5 N of Japanese = 193 F = 141, M = 53 N of Koreans = 181 F = 70, M = 111) Ethnic status: East Asian	Osteophytes, erosion, flattening of TMJ bone Interview, CT, MRI, Panoramic Radiograph	APOL3 APP CCDC81 EXT2 FRMD4A FSTL4 LOC100506 274 MRC2 N4BP1 OXR1 PCSK5 SLC24A4 THRB TPSAN9 ULK4 ZNF618	All the genes were found to be significantly associated. TSPAN9 showed strongest association (P = 8.1 × 10^−6^)	Small sample size, age and gender differed between Japanese and Korean data sets
Etoz [20] 2006 Turkey	Mean Age of Cases 21.1 ± 12.1 N = 98; F = 75, M = 23 Mean Age of Controls 22.9 ± 10.1 N = 132: F = 104, M = 28 Ethnic Status: Turkish	Difficulty or pain while opening mouth, locking of jaw, pain, noises, unusual bite, frequent headache, MRI findings-NR Anamnestic Questionnaire clinical investigation, MRI	TNF	TNFα-308GA polymorphism is not associated with TMD	NR No radiological diagnosis
Mutlu [16] 2005 Turkey	Mean Age of Cases: 22.8 ± 3.6 years; N = 93; F = 73, M = 20 Mean Age of Controls 21.10 ± 3.7 N = 91: F = 61, M = 30 Ethnic Status: NR	Difficulty or pain while opening mouth, locking of jaw, pain, noises, unusual bite, frequent headache, MRI findings-NR Anamnestic Questionnaire clinical investigation, MRI	MAOA	No evidence to support the involvement of MAOA gene in TMD	NR No radiological diagnosis

NR: Not Reported; RA: Rheumatoid Arthritis; MCTD: Mixed Connective Tissue disease; AS: Ankylosing Spondylitis; SPA: Spondyloarthropathy; ARA: American Rheumatism Association; MNYC: Modified New York Criteria; ESSG: European Spondyloarthropathy Study Group; ID: Internal Derangement; OA: Osteoarthritis; MPDS: Myofacial Pain Dysfunction Syndrome; MRI: Magnetic Resonance Imaging; CT: Computed Tomography.

**Table 2:** Animal studies.

Author publication year	Animal studies	TMD condition method of inducing	Gene studied	Results	Study limitations
Jing [25] 2014	Mice	TMD Mutant condylar cartilage Conditional Osxknockout (cKO) mice were generated by crossing Osx-loxP mice to Aggrecan-Cre mice. Cre activity induced by Tamoxifen injection	Osx	Defect in coupling chondrogenesis and osteogenesis in cKO mice, calcified cartilage in hypertrophic zone, few signs of endochondral bone formation, disorganized intramembranous bone	NR
Ishizuka [26] 2014	Mice	OA-like changes Mechanical stress	Samp8	Abnormal condylar organization, condylar degeneration, decreased chondroprogenitor cell proliferation and increased cell death	NR
Li [27] 2014	Mice	Disc Disorder Knock-in mouse line with replacement of mouse Shox2 by the human SHOX coding sequence	Shox2	Genetic association with congenital articular disc degeneration, suggesting that SHOX2 represents a susceptible locus for OA of the TMJ	NR
Jiao [28] 2014	Mice	Cartilage degradation Green fluorescent protein mice were crossed with Cre mice	Tgfb1	Abnormalities in the subchondral bone which induced cartilage degradation	Small sample size
Inman [29] 2013	Mice	Syngnathia, agenesis of TMJ Crossbreeding	Foxc1	Foxc1−/− mutant mice exhibit bilateral fusion of the upper jaw zygomatic complex to the dentary bone (syngnathia)	NR
Ricks [30] 2013	Mice	OA Heterozygous mice (Dmm/+) of a C_3_H strain were crossed to produce wildtype(+/+) and Dmm/+ mice	Col2a1	TMJ in Dmm/+ mice displayed premature articular cartilage and greater defects in chondrocyte arrangement, known biomarkers of OA were significantly expressed (P < 0.01)	NR
Yasuda [31] 2012	Mice	Abnormal endochondral ossification and Class 3 dental malocclusion, shortening of cranial base Knock-in mutation in exon 7 of Fgfr3 gene	Fgfr3	Articular disc fused with temporal bone, articular surface developed fissures, defects in endochondral ossification, abnormal glenoid fossa, defective trabecular bone formation	NR
Purcell [32] 2012	Mice	NR Inactivation of genes Spry1, Spry2	Spry1 Spry2	Combined inactivation of Spry1 and Spry2 genes leads to absence of glenoid fossa and overgrowth of lateral pterygoid and temporalis muscles	NR
Huang [13] 2011	Mice	Internal derangement Commercially bred *ank/ank* mutant mice were purchased	Ank	Fibrous ankylosis, narrower and/or ankylosed superior and inferior synovial cavities filled with fibrous connective tissue throughout the entire joint space	Utilization of 3-to-5 month old mice in this study might be responsible for the absence of erosive changes in the TMJ.
Embree [33] 2011	Mice	OA Differential gene expression analysis was performed with RNA extracted from 3-week old WT and *bgn*−/+; *fmod*−/−	4833416E15Rik Aebp1, Ahsp Angptl7, Arsk Bace1, Bgn Cartpt, Col2a1 Col9a1, Col9a3 Fmod, Hapln1 LOC 344564 Matn3, Mrpl30 Pfn1, Ptprv Rps19, Sfrp1 Slc4a1, Tspan33	The microarray analysis discovered 22 genes differentially expressed in *bgn*−/+; *fmod* −/− mouse model that could be involved in disease initiation. 5 genes (Cartpt, Sfrp1, Arsk, Slc4a1, Ptprv) changed in the mouse models and are related to osteoclast f unction/differentiation and bone turnover	NR
Purcell [34] 2009	Mice	Targeted disruption of the Gli2 zinc finger domain (Gli2^zfd/zfd^) Crossbreeding	Gli2	TMJ disk was missing, small condyle, cellular organization of growth plate was lost	NR
Gu [35] 2008	Mice	Inactivation of Shox2 Shox2 conventional knock out mice and mice with floxed Shox2 allele were generated. Wnt1-Cre;Shox2^F/F^ obtained by mating	Shox2	Dysplasia of glenoid fossa, congenital TMJ ankylosis	NR
Shibukawa [36] 2006	Mice	Absence of articular disc Absence of joint cavities Crossbreeding and induced mutation	Ihh	Mandible development was defective, condyle zonal architecture was abnormal, complete absence of functional disc and joint cavity	NR
Meng [37] 2005	Mice	OA Surgical lesion on discs, gene expression profile	Angpt12, Aqp3 Baalc, Casr, Cav, Chad, Cldn11, Cls Clu, Crabp2 Csrp2, Dkk3 Dpt, Egln3 Eln, Gda, Gda, Hig1 Hspca, Htr2a Igfbp5, Igfbp6 Il11ra1, Lg11 Lg11, Lib Meox2, Mmp3 Nb11, Nov Nr1d1, Nt5 Octnl, Plat Prelp, Prrx2 Pthlh, Scrg1 Serpina1, Sfrp4 Sod3, Spin2c Spp2, Tgfbi Thbs4, Tnfrsf11b Tnmd	Swelling, superficial fibrillation, early osteoarthritic changes	NR
Xu [38] 2003	Mice	Cartilage Degeneration Heterozygous *cho/+* mutant mice	cho	At age 6 months OA-like changes became more severe, including flattening of the condylar head, loss of proteoglycans, and a reduced hypertrophic zone	NR
Gu [39] 2014	Mice	Apoptotic cells *Wnt*1*-Cre; Bmpr*1*a^F/F^* embryos obtained by crossing *Wnt*1*-Cre; Bmpr*1*a^F/+^* with *Bmpr*1*a^F/F^* line	Bmpr1a	Agenesis Failed formation of functional fibrocartilage layer, Failure of disc separation from hypoplastic condyle	NR
Li [40] 2014	Mice	Dysplasia Cross of Wnt-Cre mice with *pMes-stopShox*2 mice	Shox2	Increased number of apoptotic cells in the glenoid fossa causing glenoid fossa dysplasia, dysplasia of condyle	NR
Wang [41] 2014	Mice	TMJ Cartilage Degeneration Crossbreeding to generate *β-catenin*(*ex*3)*^Col2ER^*, Tamoxifen administered	Ctnnb1	Significant reduction in TMJ joint space, cartilage thickness was significantly decreased, increase in cartilage degrading enzymes, OA-like phenotype	NR
Meng [42] 2007	Rats	OA Surgically, lesions created in discs	Aqp1 Aqp3	High expression and different localization of AQP3 in OA cartilage. No significant difference between OA and normal controls	NR
Yu [43] 2012	Rats	Condyle cartilage Degeneration Occlusal treatment carried out to induce degenerative changes in the mandibular condylar cartilage	Igf1 Gfr1 Igfbp3	Obvious OA-like changes were observed in 2 week female experimental group than those in control group (P < 0.01) and 2 week male experimental group (P < 0.05), Expression of IGF1 in the 2 week females was lower than males (P < 0.01), IGFR1 was significantly lower in 2 week female (P < 0.05) but increased in 2 week male experimental group, IGFBP3 in all female subgroups was significantly lower than that in their male counterparts	Differences in occlusion between rats and humans
Asakawa-Tanne [44] 2015	Porcine	Disc indentation Porcine cranial heads were subjected to cyclic loading	Cox2 Il1b Mmp1 Mmp3 Mmp9	Compromised lubrication in TMJ is associated with altered frictional properties and surface wear of condylar cartilage	Amount and nature of loading used in the study do not represent the actual TMJ dynamics *in vivo*
Ge [45] 2009	Rabbit	Cartilage destruction Primary rabbit condylar chondrocytes were treated with IL-*β*, purified WNT5A protein, or both and transfected with Wnt5a expression vector	Wnt5a	WNT5A is associated with cartilage destruction by promoting expression of MMP1, MMP3, MMP9, MMP13	NR

NR: Not reported; OA: Osteoarthritis.

## Abbreviations

DC/TMD: Diagnostic Criteria for TMD
ESR1: Estrogen Receptor Alpha
GWAS: Genome-Wide Association Study
MAOA: Monoamine Oxidase-A
NSAIDs: Non-Steroidal Anti-Inflammatory Drugs
PRISMA: Preferred Reporting Items for Systematic Reviews and Meta-Analyses
RDC/TMD: Research Diagnostic Criteria for Temporomandibular Disorders
TMDs: Temporomandibular Joint Disorders
TMJ: Temporomandibular Joint
TNF: Tumor Necrosis Factor Alpha
