# Supplementary material for: Gene Mutations Associated with Temporomandibular Joint Disorders: A Systematic Review
Source: OAlib. Author manuscript; Available in PMC 2016 Sep 30. (PMC5045035; doi:10.4236/oalib.1101583)
Supplement: Supplementary file 1 [file NIHMS784630-supplement-supplement_1.pdf]

Supplemental Information

Table S1. Summary of databases searched.

Table	Vendor/ Interface	Database	Date searched	Database update	Searcher(s)
1a	Ovid	Medline®	2/25/2015	1996 to February Week 3 2015; In-Process & Other Non-Indexed Citations February 24, 2015	Helena M. VonVille; Dhruvee Sangani
1b	National Library of Medicine	PubMed	1/28/2015	1/28/2015	Helena M. VonVille; Dhruvee Sangani
1c	Ovid	Embase®	1/29/2015	1974 to 2015 January 27	Helena M. VonVille

Table S2. Codebook.

ID	Variable name (coding instructions)	Values, text codes
	**Citation information**	
C 1	**Ref ID**	**(Numerical) (ranges from 1-1010)**
C 2	**Name of coder**	
C 3	**Publication date**	**YYYY (ranges from 2000-2015)**
C 4	**Author**	
C 5	**Type of citation**	Full text (from journal article)
C 7	**Secondary cite(s)-** Ref ID #, publication date, author, journal	
C8	**Number of studies reported in this citation**	Note: default = 1
	**Study level information (characteristics and results)**	
	Study design	Laboratory experiment/translational research<br>Other (specify):
S 3	**Study location-state/province, city, & country:**	
S 4	**Sponsor** (check one)	Industry sponsored<br>Cannot be ascertained<br>Other (specify)
S 5	**Type of TMD disorder** alphabetize list	Agenesis<br>Ankylosis<br>Disc derangement<br>Dysplasia<br>Internal derangement<br>Osteoarthritis<br>Syngnathia<br>Other (specify)
S 6	**Species**	Humans<br>Animals a) Mice; b) Rabbit; c) Rat; d) Sheep Other (Specify)
S 7	**Gene(s) studied**	List out all genes
	**Quality level information**	

Continued

Q 1	**Did the study describe the method of embryo collection and genotyping?**	**1-Yes<br>2-No<br>3-NA**
Q 2	**Did the study describe eligibility criteria and methods used to select participants?**	**1-Yes<br>2-No<br>3-NA**
Q 3	**Were the statistical analysis reported?**	**1-Yes<br>2-No<br>3-NA**
Q 4	**Did the study describe the trial design?**	**1-Yes<br>2-No<br>3-NA**
Q 5	**Did the study explain sample size calculations**	**1-Yes<br>2-No<br>3-NA**
Q 7	**Did the study describe blinding?**	**1-Yes<br>2-No<br>3-NA**
Q 8	**What were the key limitations according to the authors?**	
Q9	**Did the authors discuss the external validity?**	**1-Yes<br>2-No<br>3-NA**
Q 10	**Did the study describe eligibility criteria and methods used to select participants?**	**1-Yes<br>2-No<br>3-NA**
Q 12	**Was the association of genes with TMJ Disorders clearly described?**	**1-Yes<br>2-No<br>3-NA**
Q 13	**Was the study outcome defined?**	**1-Yes<br>2-No<br>3-NA**
Q 14	**Did the study describe any measures to minimize potential sources of bias?**	**1-Yes<br>2-No<br>3-NA**
Q15	**Did the study explain how was sample size arrived at?**	**1-Yes<br>2-No<br>3-NA**
Q 18	**What were the key limitations according to the authors?**	
